# Effect of Obesity on the Exposure of Long-acting Cabotegravir and Rilpivirine: A Modeling Study

**DOI:** 10.1093/cid/ciae060

**Published:** 2024-02-03

**Authors:** Sara Bettonte, Mattia Berton, Felix Stader, Manuel Battegay, Catia Marzolini

**Affiliations:** Division of Infectious Diseases and Hospital Epidemiology, Departments of Medicine and Clinical Research, University Hospital Basel, Basel, Switzerland; Faculty of Medicine, University of Basel, Basel, Switzerland; Division of Infectious Diseases and Hospital Epidemiology, Departments of Medicine and Clinical Research, University Hospital Basel, Basel, Switzerland; Faculty of Medicine, University of Basel, Basel, Switzerland; Certara UK Limited, Sheffield, United Kingdom; Division of Infectious Diseases and Hospital Epidemiology, Departments of Medicine and Clinical Research, University Hospital Basel, Basel, Switzerland; Faculty of Medicine, University of Basel, Basel, Switzerland; Division of Infectious Diseases and Hospital Epidemiology, Departments of Medicine and Clinical Research, University Hospital Basel, Basel, Switzerland; Faculty of Medicine, University of Basel, Basel, Switzerland; Department of Molecular and Clinical Pharmacology, University of Liverpool, Liverpool, United Kingdom; Service and Laboratory of Clinical Pharmacology, Department of Laboratory Medicine and Pathology, University Hospital Lausanne and University of Lausanne, Lausanne, Switzerland

**Keywords:** obesity, PBPK modeling, long-acting, cabotegravir, rilpivirine

## Abstract

**Background:**

Obesity is increasingly prevalent among people with human immunodeficiency virus (HIV, PWH). Obesity can reduce drug exposure; however, limited data are available for long-acting (LA) antiretrovirals. We performed in silico trials using physiologically based pharmacokinetic (PBPK) modeling to determine the effect of obesity on the exposure of LA cabotegravir and rilpivirine after the initial injection and after multiple injections.

**Methods:**

Our PBPK model was verified against available clinical data for LA cabotegravir and rilpivirine in normal weight/ overweight (body mass index [BMI] <30 kg/m^2^) and in obese (BMI >30 kg/m^2^). Cohorts of virtual individuals were generated to simulate the exposure of LA cabotegravir/rilpivirine up to a BMI of 60 kg/m^2^. The fold change in LA cabotegravir and rilpivirine exposures (area under the curve [AUC]) and trough concentrations (C_min_) for monthly and bimonthly administration were calculated for various BMI categories relative to normal weight (18.5–25 kg/m^2^).

**Results:**

Obesity was predicted to impact more cabotegravir than rilpivirine with a decrease in cabotegravir AUC and C_min_ of >35% for BMI >35 kg/m^2^ and in rilpivirine AUC and C_min_ of >18% for BMI >40 kg/m^2^ at steady-state. A significant proportion of morbidly obese individuals were predicted to have both cabotegravir and rilpivirine C_min_ below the target concentration at steady-state with the bimonthly administration, but this was less frequent with the monthly administration.

**Conclusions:**

Morbidly obese PWH are at risk of presenting suboptimal C_min_ for cabotegravir/rilpivirine after the first injection but also at steady-state particularly with the bimonthly administration. Therapeutic drug monitoring is advised to guide dosing interval adjustment.

## Teaser

Obesity reduces the exposure of long-acting cabotegravir/rilpivirine with a significant number of morbidly obese individuals predicted to have concentrations below the target thresholds at steady-state notably for the bimonthly administration. Therapeutic drug monitoring is advised to guide dosing interval adjustment.

The World Health Organization (WHO) defines obesity as an excessive fat accumulation that can potentially impair health [1]. Body mass index (BMI) is the body size descriptor most commonly used to classify overweight (ie, BMI 25–30 kg/m^2^), obese (ie, BMI 30–40 kg/m^2^), and morbidly obese (ie, BMI >40 kg/m^2^) individuals.

The prevalence of obesity tripled between 1975 and 2016, with over 4 million people dying of obesity every year [1]. The developing countries are not spared by this epidemic as the prevalence of obesity ranges from 14% to 31% [2]. Thanks to highly effective antiretrovirals (ARVs) which led to improved health, the obesity trend in people with human immunodeficiency virus (HIV, PWH) is nowadays similar to that of the general population [3, 4]. In 2019, approximately 20% of the participants enrolled in the Swiss HIV Cohort Study were obese [4]. Furthermore, the percentage of overweight/obese increased from 28% in 1985–1990 to 51% in 1996–2004 in the United States [5, 6]. Obesity related physiological changes, leading notably to an increase in cardiac output and related increase in liver and renal blood flows, can potentially lower drug exposure [7–9]. However, pharmacokinetic data are often limited given that obese individuals are generally excluded from clinical trials [10].

Physiologically based pharmacokinetic (PBPK) modeling is a mathematical tool that simulates the pharmacokinetics (PK) of a compound by combining the drug properties and the physiology of the population of interest. PBPK is an approach accepted by health authorities and can be used to study unknown clinical scenarios in special populations [11, 12]. PBPK modeling was previously used to evaluate the impact of obesity on the drug disposition of several oral ARVs [8] and to investigate drug-drug interaction (DDI) scenario in obese individuals [13].

The approval of long-acting (LA) drugs for the treatment and prevention of HIV raised also the question of the exposure in obese PWH. Even though LA formulations have been used for decades for the treatment of schizophrenia and for contraception, few studies have characterized the pharmacokinetics in obese individuals. Lower paliperidone plasma concentrations have been reported after the initial injection in overweight/obese individuals compared to normal weight individuals. However, at steady-state, paliperidone exposure was comparable in obese and non-obese individuals [14]. Similarly, the concentrations of LA cabotegravir at the end of the dosing interval have been shown to be lower in obese (BMI ≥30 kg/m^2^) compared to non-obese (BMI <30 kg/m^2^) individuals after the first injection and to align once steady-state is reached [15, 16].

The use of a longer needle (2-inch needle) in obese individuals was shown to significantly increase the trough concentration (C_min_) of LA cabotegravir after the initial injection, as it helps to reach the muscle rather than the subcutaneous adipose tissue, where blood flow is reduced which could result in lower initial concentrations [16]. Data from the FLAIR, ATLAS, and ATLAS-2 M studies have also shown that LA cabotegravir and rilpivirine could be impacted differently by obesity as, unlike cabotegravir, rilpivirine concentrations were comparable between normal weight/overweight and obese individuals after the initial injection [16], which could possibly be explained by differences in the physicochemical properties. To date, no study has thoroughly assessed the impact of obesity on the pharmacokinetics of LA cabotegravir/rilpivirine for different obesity categories, particularly for morbidly obese (BMI ≥40 kg/m^2^). The obese individuals enrolled in the phase III trials had indeed an overall median BMI of 33 kg/m^2^ and therefore are not representative of the morbidly obese individuals. Thus, the aim of this study was to conduct in-silico trials using PBPK modeling to assess the effect of obesity up to a BMI of 60 kg/m^2^ on the exposure of LA cabotegravir and rilpivirine.

## METHODS

Three main steps were taken to verify our in-house PBPK model [17, 18] and evaluate the effect of obesity on the exposure of LA cabotegravir/rilpivirine. First, the developed drug models for cabotegravir and rilpivirine were verified against available observed data after intramuscular administration for normal weight/overweight (BMI <30 kg/m^2^) and obese (BMI ≥30 kg/m^2^) individuals at steady-state. Second, we simulated the exposure of LA cabotegravir/rilpivirine administered monthly or every other month for various BMI categories (ie, BMI 25–30 kg/m^2^, 30–35 kg/m^2^, 35–40 kg/m^2^, 40–50 kg/m^2^, 50–60 kg/m^2^). Third, we calculated the fold change for each BMI categories relative to the normal weight individuals (BMI 18.5–25 kg/m^2^).

### PBPK Model Verification in Normal Weight and Obese Individuals

Our in-house whole-body PBPK model developed in Matlab®2020a [17] was implemented with a framework describing the release of the LA ARVs from the depot upon intramuscular administration [18]. The PBPK model was informed with our previously validated equations describing the physiological and anatomical changes of an obese population with a BMI range between 18.5 and 60 kg/m^2^ and aged between 20 and 50 years [10]. The ability of the PBPK model to predict the pharmacokinetics in normal weight/overweight (<30 kg/m^2^) and in obese (≥30 kg/m^2^) individuals after oral administration was demonstrated in our previous work [8]. For the purpose of this study, we verified also that the model can predict intramuscular cabotegravir and rilpivirine drug concentrations in obese individuals by matching the study design of Phase III clinical studies (ATLAS, FLAIR, and ATLAS-2 M), including the demographic parameters (ie, age range, proportion of female, BMI range) and the dosing regimen [16]. The drug models were considered verified when the simulated median C_min_ at weeks 8 and 48 were within 2-fold of clinical observed data as described in PBPK model guidelines [19, 20]. The equations, the properties of cabotegravir/rilpivirine to inform the PBPK model [21, 22], as well as the predictive performance of the model for oral rilpivirine and cabotegravir pharmacokinetics are presented in the supplementary material (Supplementary Tables 1 and 2).

### Impact of Obesity on the Exposure of LA Cabotegravir and Rilpivirine

The verified drug models were then used to simulate the exposure of LA cabotegravir/rilpivirine administered monthly or every other month for various BMI categories (ie, BMI 18.5–25 kg/m^2^, BMI 25–30 kg/m^2^, 30–35 kg/m^2^, 35–40 kg/m^2^, 40–50 kg/m^2^, 50–60 kg/m^2^). Six cohorts of 100 virtual individuals aged 20–50 years (50% female) were generated for each BMI category, and the design of the ATLAS/ FLAIR and ATLAS-2 M was reproduced for the in-silico trials [23–25]. Subsequently, the fold change in the area under the concentration-time curve (AUC) and C_min_ at steady-state (week 96) was calculated for the different BMI categories relative to normal weight virtual individuals (BMI 18.5–25 kg/m^2^). Additionally, the percentage of virtual individuals below the protein adjusted 90% inhibitory concentration (PA-IC_90_) (ie, 166 ng/mL [26]) and below the 4-fold PA-IC_90_ (ie, 664 ng/mL [26]) for LA cabotegravir were calculated at week 8, 48, and 96 both for the monthly and bimonthly administration. Similarly, for LA rilpivirine, the percentage of virtual individuals below the 25th percentile (ie, 32 ng/mL [27]) and below the minimal clinical concentration for therapeutic response (ie, 50 ng/mL [28]) were calculated.

## RESULTS

### PBPK Model Verification in Normal Weight and Obese Individuals

**Figure 1.**
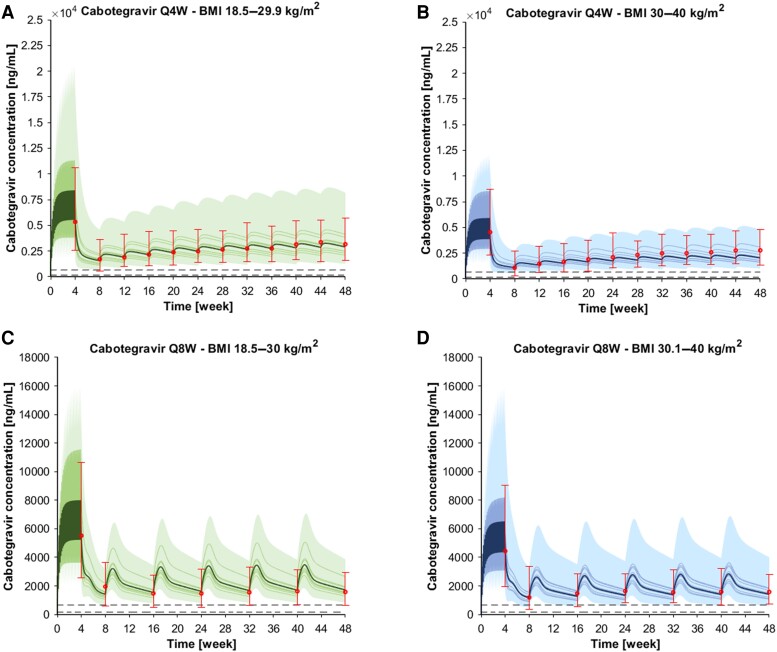
Concentration-time profiles for cabotegravir after an oral daily 30 mg dose for 1 month, one 600 mg IM loading dose, and multiple 400 mg maintenance doses administered every month (Q4W) in (*A*) normal weight individuals (BMI 18.5–29.9 kg/m^2^) and (*B*) obese individuals (BMI 30–40 kg/m^2^). Concentration-time profiles for cabotegravir after an oral daily 30 mg dose for one month, one 600 mg IM loading dose, and multiple 600 mg maintenance doses administered every other month (Q8W) in (*C*) normal weight individuals (BMI 18.5–30 kg/m^2^) and (*D*) obese individuals (BMI 30–40 kg/m^2^). The solid lines, the solid bold line, and the shaded area represent the geometric mean of each in silico trial, the geometric mean of all trials, and the 90% normal range of all virtual individuals. The red markers represented the median of the clinically observed data, the 95th and the 5th percentile. The dashed lines represent the PA-IC_90_ (ie, 166 ng/mL) and the 4-fold PA-IC_90_ (ie, 664 ng/mL). Abbreviations: BMI, body mass index; Q4W, administered every 4 wks; Q8W, administered every 8 wks; wks, weeks.

**Figure 2.**
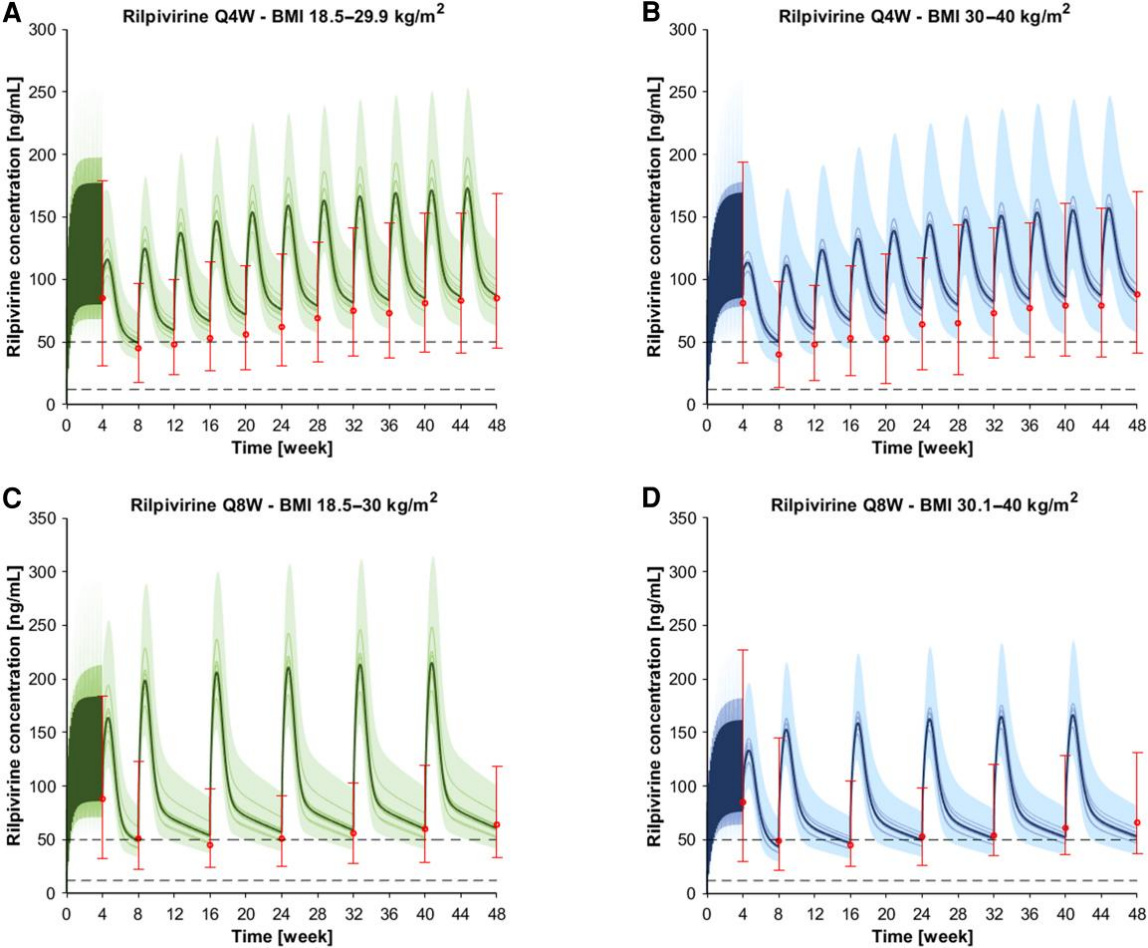
Concentration-time profiles for rilpivirine after an oral daily 25 mg dose for 1 month, 1 900 mg IM loading dose, and multiple 600 mg maintenance doses administered every month (Q4W) in (*A*) normal weight individuals (BMI 18.5–29.9 kg/m^2^) and (*B*) obese individuals (BMI 30–40 kg/m^2^). Concentration-time profiles for rilpivirine after an oral daily 25 mg dose for one month, one 900 mg IM loading dose, and multiple 900 mg maintenance doses administered every other month (Q8W) in (*C*) normal weight individuals (BMI 18.5–30 kg/m^2^) and (*D*) obese individuals (BMI 30–40 kg/m^2^). The solid lines, the solid bold line, and the shaded area represent the geometric mean of each in silico trial, the geometric mean of all trials, and the 90% normal range of all virtual individuals. The red markers represented the median of the clinically observed data, the 95th and the 5th percentile. The dashed lines represent the 5th percentile (ie, 32 ng/mL) and the minimal concentration for therapeutic response (ie, 50 ng/mL). Abbreviations: Q4W, administered every 4 wks; Q8W, administered every 8 wks; wks, weeks.

The drug model for LA cabotegravir was successfully verified for the monthly and the bimonthly administration as the simulations were within 1.5-fold of the clinical observed data (Figure 1). Specifically, the obese:normal weight ratio for C_min(8week)_ and C_min(48week)_ were 1.13 and 0.81, respectively, for the monthly administration and 1.33 and 0.83, respectively, for the bimonthly administration (Table 1). Similarly, the drug model for LA rilpivirine was successfully verified against clinical observed data for the monthly and the bimonthly administration. The simulations were within 1.25-fold of the clinical observed data (Figure 2). Specifically, the obese:normal weight ratio for C_min(8week)_ and C_min(48week)_ were 1.13 and 0.95, respectively, for the monthly administration and 0.92 and 0.84, respectively, for the bimonthly administration (Table 1).

**Table 1.** Predicted Versus Observed Data in Obese Versus Normal Weight Individuals

Cabotegravir Q4W									
	Age: 20–50 y, Female: 23% BMI: 18.5–29.9 kg/m^2^			Age: 23–50 y, Female: 42% BMI: 30–40 kg/m^2^			Ratio Obese/Normal Weight		
Dosing time	Observed	Predicted	Ratio P/O	Observed	Predicted	Ratio P/O	Observed	Predicted	Ratio P/O
C_min_ 8 wk [ng/mL]	1712	1578	0.92	1085	1132	1.04	0.63	0.72	1.13
C_min_ 48 wk [ng/mL]	3162	2955	0.93	2776	2105	0.76	0.88	0.71	0.81

Cabotegravir Q8W									
	Age: 20–50 y, Female: 18% BMI: 18.5–30 kg/m^2^			Age: 32–50 y, Female: 42% BMI: 30–40 kg/m^2^			Ratio Obese/ Normal Weight		
Dosing time	Observed	Predicted	Ratio P/O	Observed	Predicted	Ratio P/O	Observed	Predicted	Ratio P/O
C_min_ 8 wk [ng/mL]	1952	1324	0.68	1194	1080	0.90	0.61	0.82	1.33
C_min_ 48 wk [ng/mL]	1576	1595	1.01	1576	1319	0.84	1.00	0.83	0.83

Rilpivirine Q4W									
	Age: 20–50 y, Female: 23% BMI: 18.5–29.9 kg/m^2^			Age: 23–50 y, Female: 42% BMI: 30–40 kg/m^2^			Ratio Obese/ Normal Weight		
Dosing time	Observed	Predicted	Ratio P/O	Observed	Predicted	Ratio P/O	Observed	Predicted	Ratio P/O
C_min_ 8 wk [ng/mL]	45	48	1.07	40	48	1.20	0.89	1.00	1.13
C_min_ 48 wk [ng/mL]	85	85	1.00	88	84	0.95	1.04	0.99	0.95

Rilpivirine Q8W									
	Age: 20–50 y, Female: 18% BMI: 18.5–30 kg/m^2^			Age: 32–50 y, Female: 42% BMI 30–40 kg/m^2^			Ratio Obese/Normal Weight		
Dosing time	Observed	Predicted	Ratio P/O	Observed	Predicted	Ratio P/O	Observed	Predicted	Ratio P/O
C_min_ 8 wk [ng/mL]	51	48	0.94	49	42	0.86	0.96	0.88	0.92
C_min_ 48 wk [ng/mL]	64	60	0.94	66	52	0.79	1.03	0.87	0.84

The results are represented as median.

Abbreviations: BMI, body mass index; C_min_, trough concentration; O, observed data; P, predicted data; Q4W, administered every 4 wks; Q8W, administered every 8 wks; wk, week.

### Impact of Obesity on the Exposure of LA Cabotegravir and Rilpivirine

#### Cabotegravir

**Figure 3.**
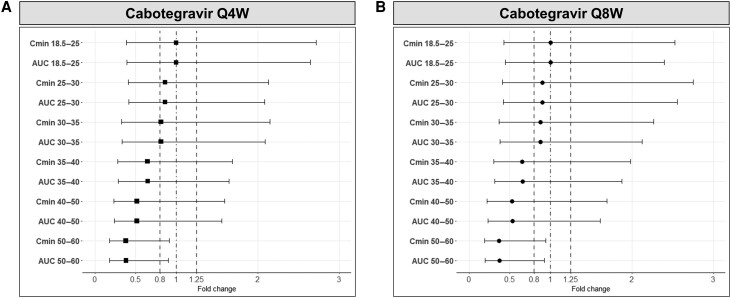
Fold change in trough concentration (C_min_) and exposure (at steady-state administration) for different BMI categories relative to normal weight virtual individuals (18.5–25 kg/m^2^) for LA cabotegravir administered (*A*) monthly (Q4W) and (*B*) every other month (Q8W). The results are expressed as geometric mean, 5th percentile, and 95th percentile. The dot dash line represents the unit line, and the dashed lines represent the bioequivalence range (0.8–1.25). Abbreviations: AUC, area under the concentration-time curve; C_min_, trough concentration; Q4W, monthly administration; Q8W, administration every other month.

LA cabotegravir requires 44 weeks to reach steady-state [29]; thus the fold change in C_min_ and AUC for each BMI categories relative to normal weight were calculated at week 96. Specifically, for LA cabotegravir administered monthly, C_min_ and AUC were predicted to be decreased by 14% in virtual overweight individuals (ie, BMI 25–30 kg/m^2^) and by 19% in virtual obese individuals (ie, 30–35 kg/m^2^). Despite this reduction, C_min_ and AUC were predicted to be still within the bioequivalence range (0.8–1.25) (Figure 3*A*). On the other hand, a more pronounced effect was predicted in individuals with higher BMI with a reduction in C_min_ and AUC of 35% for the BMI category of 35–40 kg/m^2^, 48% for the BMI category of 40–50 kg/m^2^, and 62% for the BMI of 50–60 kg/m^2^ (Figure 3*A*). A similar reduction was predicted when LA cabotegravir was administered every other month. The reduction in C_min_ and AUC were within the bioequivalence range (0.8–1.25) for overweight (10%) and for obese (12%) individuals (Figure 3*B*), whereas the decrease was predicted to be 35% for BMI of 35–40 kg/m^2^, 47% for BMI of 40–50 kg/m^2^, and 63% for BMI of 50–60 kg/m^2^ (Figure 3*B*).

For LA cabotegravir administered monthly, none of the virtual individuals in the various BMI categories were predicted to be below the PA-IC_90_ (Table 2). However, the percentage of individuals predicted to be below the 4-fold PA-IC_90_ was significant for all BMI categories particularly at week 8 after the first injection but less frequent at week 48 and 96 after multiple injections (Table 2). Specifically, at week 8, 54% of virtual individuals having a BMI of 50–60 kg/m^2^ were below the 4-fold PA-IC_90_ (ie, 664 ng/mL [26]), whereas at week 48 and 96 only 11% and 9%, respectively, were below this threshold (Table 2). Similarly, when LA cabotegravir was administered bimonthly, none of the virtual individuals were predicted to be below the PA-IC_90_; however, the percentage of virtual individuals below the 4-fold PA-IC_90_ (ie, 664 ng/mL [26]) increased substantially with increasing BMI (Table 2). Specifically, 66% of virtual individuals with a BMI of 50–60 kg/m^2^ were below the 4-fold PA-IC_90_ at week 8, and 50% were still below this threshold at weeks 48 and 96 (Table 2).

**Table 2.** Percentage of Virtual Individuals Below the PA-IC_90_ (ie, 166 ng/mL) [25] and the 4-fold PA-IC_90_ (ie, 664 ng/mL) [25] for the Different BMI Groups at Various Time Points After the Initiation of LA Cabotegravir

BMI Group	Time Point	Sampling Condition	Percentage of Virtual Individuals With Trough Plasma Concentration Below 166 ng/mL	Percentage of Virtual individuals With Trough Plasma Concentration Below 664 ng/mL
Cabotegravir Q4W				
18.5–25 kg/m^2^	8 wk	Pre-dose	0	6
	48 wk	Pre-dose	0	0
	96 wk	Pre-dose	0	0
25–30 kg/m^2^	8 wk	Pre-dose	0	4
	48 wk	Pre-dose	0	0
	96 wk	Pre-dose	0	0
30–35 kg/m^2^	8 wk	Pre-dose	0	12
	48 wk	Pre-dose	0	0
	96 wk	Pre-dose	0	0
35–40 kg/m^2^	8 wk	Pre-dose	0	19
	48 wk	Pre-dose	0	0
	96 wk	Pre-dose	0	0
40–50 kg/m^2^	8 wk	Pre-dose	0	36
	48 wk	Pre-dose	0	2
	96 wk	Pre-dose	0	0
50–60 kg/m^2^	8 wk	Pre-dose	0	54
	48 wk	Pre-dose	0	11
	96 wk	Pre-dose	0	9
Cabotegravir Q8W				
18.5–25 kg/m^2^	8 wk	Pre-dose	0	4
	48 wk	Pre-dose	0	2
	96 wk	Pre-dose	0	2
25–30 kg/m^2^	8 wk	Pre-dose	0	6
	48 wk	Pre-dose	0	1
	96 wk	Pre-dose	0	1
30–35 kg/m^2^	8 wk	Pre-dose	0	11
	48 wk	Pre-dose	0	2
	96 wk	Pre-dose	0	2
35–40 kg/m^2^	8 wk	Pre-dose	0	21
	48 wk	Pre-dose	0	10
	96 wk	Pre-dose	0	10
40–50 kg/m^2^	8 wk	Pre-dose	0	34
	48 wk	Pre-dose	0	27
	96 wk	Pre-dose	0	25
50–60 kg/m^2^	8 wk	Pre-dose	0	66
	48 wk	Pre-dose	0	50
	96 wk	Pre-dose	0	50

Abbreviations: PA-IC_90_, protein-adjusted 90% inhibitory concentration; Q4W, administered every 4 weeks; Q8W, administered every 8 weeks; wk, week.

#### Rilpivirine

**Figure 4.**
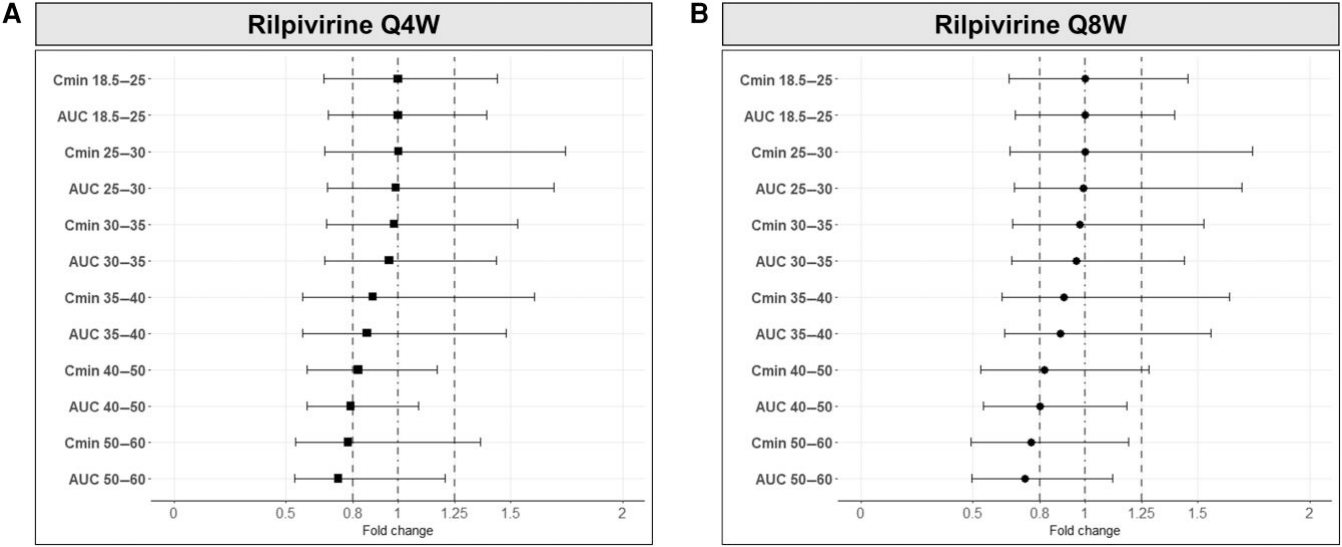
Fold change in trough concentration (C_min_) and exposure (at steady-state administration) for different BMI categories relative to normal weight virtual individuals (18.5–25 kg/m^2^) for LA rilpivirine administered (*A*) monthly (Q4W) and (*B*) every other month (Q8W). The results are expressed as geometric mean, 5th percentile, and 95th percentile. The dot-dashed line represents the unit line, and the dashed lines represent the bioequivalence range (0.8–1.25). Abbreviations: AUC, area under the concentration-time curve; C_min_, trough concentration; Q4W, monthly administration; Q8W, administration every other month.

LA rilpivirine requires 48 weeks to reach steady-state [29]; thus the fold change in C_min_ and AUC for each BMI categories relative to normal weight were also calculated at week 96. Specifically, for LA rilpivirine administered monthly, the C_min_ and AUC were predicted to be minimally impacted and to remain within the bioequivalence range (0.8–1.25) in overweight (ie, BMI 25–30 kg/m^2^) and obese (ie, BMI 30–35 kg/m^2^; BMI 35–40 kg/m^2^) individuals (Figure 4*A*). For individuals with higher BMI, C_min_ and AUC were reduced by 18% and 21%, respectively, for individuals with a BMI of 40–50 kg/m^2^ and by 22% and 27% for those with a BMI of 50–60 kg/m^2^ (figure 4*A*). Similar reductions were predicted for C_min_ and AUC when LA rilpivirine was administered every other month (Figure 4*B*).

For LA rilpivirine administered monthly, a small percentage of individuals were predicted to be below the 25th percentile (ie, 32 ng/mL [27]) only after the first injection at week 8 (Table 3). On the other hand, the number of individuals below the minimal clinical concentration for therapeutic response (ie, 50 ng/mL [28]) was >70% at week 8 for individuals with BMI ≥35 kg/m^2^ (Table 3). However, most individuals were predicted to have rilpivirine C_min_ above this threshold at week 48 and 96 (Table 3). Similar results were obtained when LA rilpivirine was administered every other month with individuals below the 25th percentile (ie, 32 ng/mL [27]) mostly at week 8 (Table 3). On the other hand, the percentage of individuals below the minimal clinical concentration for therapeutic response (ie, 50 ng/mL [28]) was >50% at week 8 for all BMI categories (Table 3) and it remained above this percentage at weeks 48 and 96 for individuals with a BMI ≥40 kg/m^2^ (Table 3).

**Table 3.** Percentage of Virtual Individuals Below 25th Percentile (ie, 32 ng/mL) [26] and Below the Minimal Clinical Concentration for Therapeutic Response (ie, 50 ng/mL) [27] for the Different BMI Groups at Various Time Points After the Initiation of LA Rilpivirine

BMI Group	Time Point	Sampling Condition	Percentage of Virtual Individuals With Trough Plasma Concentration Below 32 ng/mL	Percentage of Virtual Individuals With Trough Plasma Concentration Below 50 ng/mL
Rilpivirine Q4W				
18.5–25 kg/m^2^	8 wk	Pre-dose	2	42
	48 wk	Pre-dose	0	1
	96 wk	Pre-dose	0	1
25–30 kg/m^2^	8 wk	Pre-dose	1	51
	48 wk	Pre-dose	0	0
	96 wk	Pre-dose	0	0
30–35 kg/m^2^	8 wk	Pre-dose	1	49
	48 wk	Pre-dose	0	0
	96 wk	Pre-dose	0	0
35–40 kg/m^2^	8 wk	Pre-dose	7	71
	48 wk	Pre-dose	0	1
	96 wk	Pre-dose	0	0
40–50 kg/m^2^	8 wk	Pre-dose	5	76
	48 wk	Pre-dose	0	1
	96 wk	Pre-dose	0	1
50–60 kg/m^2^	8 wk	Pre-dose	13	81
	48 wk	Pre-dose	0	5
	96 wk	Pre-dose	0	4
Rilpivirine Q8W				
18.5–25 kg/m^2^	8 wk	Pre-dose	5	56
	48 wk	Pre-dose	1	18
	96 wk	Pre-dose	1	17
25–30 kg/m^2^	8 wk	Pre-dose	2	65
	48 wk	Pre-dose	0	22
	96 wk	Pre-dose	0	18
30–35 kg/m^2^	8 wk	Pre-dose	2	58
	48 wk	Pre-dose	0	27
	96 wk	Pre-dose	0	25
35–40 kg/m^2^	8 wk	Pre-dose	5	69
	48 wk	Pre-dose	0	43
	96 wk	Pre-dose	0	41
40–50 kg/m^2^	8 wk	Pre-dose	15	71
	48 wk	Pre-dose	4	55
	96 wk	Pre-dose	4	48
50–60 kg/m^2^	8 wk	Pre-dose	21	76
	48 wk	Pre-dose	8	64
	96 wk	Pre-dose	7	60

Abbreviations: BMI, body mass index; Q4W, administered every 4 weeks; Q8W, administered every 8 weeks; wk, week.

In summary, obesity was predicted to impact more cabotegravir than rilpivirine with a decrease in cabotegravir AUC and C_min_ of >35% for BMI >35 kg/m^2^ and in rilpivirine AUC and C_min_ of >18% for BMI >40 kg/m^2^ at steady-state. A significant proportion of morbidly obese individuals were predicted to have both cabotegravir and rilpivirine C_min_ below the target concentration at steady-state with the bimonthly administration.

## DISCUSSION

Obesity is characterized by anatomical and physiological changes that can reduce drug exposure [10]. Using PBPK modeling combined with therapeutic drug monitoring (TDM) data from the participants of the Swiss HIV Cohort Study, we previously demonstrated that obesity reduces the exposure of contemporary oral ARVs to an extent that does not warrant a dose adjustment. Nevertheless, TDM is recommended for rilpivirine (exposure reduced by 40% for a BMI >40 kg/m^2^) and etravirine as obesity has a more pronounced effect on these agents so that morbidly individuals have a higher risk to present rilpivirine or etravirine C_min_ below the minimal concentration targets (ie, 50 ng/mL for rilpivirine [28] and 300 ng/mL for etravirine [30]) [8].

Obesity seems to be a relevant factor for LA cabotegravir/rilpivirine exposure as multivariable logistic regression analyses using Phase III data have identified obesity and low initial drug concentrations as risk factors for virological failure [27]. However, the effect of obesity on LA cabotegravir/rilpivirine pharmacokinetics has not been fully characterized as obese individuals enrolled in the Phase III trials had an overall median BMI of 33 kg/m^2^ [16], which is not representative of morbidly obese subjects. Therefore, we evaluated the effect of obesity on the exposure of intramuscular LA cabotegravir and rilpivirine for several BMI categories. Obesity was predicted to decrease the exposure of these agents mainly due to an increase in clearance as a result of enhanced cardiac output and the related increase in liver and renal blood flows with obesity [7]. We found that LA cabotegravir was more impacted by obesity compared to LA rilpivirine because, for BMI >35 kg/m^2^, the fold change in C_min_ and AUC was largely outside the bioequivalence range (0.8–1.25) for cabotegravir, whereas it remained within or close to this range for rilpivirine for all BMI categories. These findings are in line with data from the FLAIR, ATLAS and ATLAS-2 M phase III trials, which showed that LA rilpivirine C_min_ was similar in obese and non-obese individuals after the first injection whereas it was lower for LA cabotegravir in PWH with a BMI >30 kg/m^2^ [16]. This difference could possibly be explained by differences in the physicochemical properties or metabolic pathways (ie, cabotegravir is more hydrophilic and is mainly metabolized by uridine 5'-diphospho-glucuronosyltransferase (UGT) 1A1 [29]; rilpivirine is more lipophilic and undergoes mainly cytochrome (CYP) 3A4 metabolism [29]). Such differences may be important considering that with obesity, various physiological changes occur, including notably an increase in the adipose tissue, cardiac output and related increase in liver and renal blood flows. Furthermore, the serum albumin and the expression of CYP3A4 decrease with obesity, whereas the expression of UGT1A1 increases [10].

Our findings indicate that obesity reduces the exposure of intramuscular ARVs to a similar extent than oral ARVs [8]; however, the percentage of individuals predicted to be below the minimal concentration threshold is significantly higher for the intramuscular ARVs. Importantly, our simulations indicate that morbidly obese individuals are at risk of presenting suboptimal trough concentrations for cabotegravir and rilpivirine not only after the first injection but also at steady-state particularly with the bimonthly administration. This finding is supported by the data of the Dutch ATHENA cohort reporting low cabotegravir or rilpivirine concentrations in two obese PWH (BMI: 31.5 and 45.5 kg/m^2^) who had a documented viral failure 8 and 13 months after the initiation of the LA treatment [31]. Long needles were used in these individuals and none of them had other risk factors for virological failure. Our findings and these real-life data suggest that caution is needed in obese and in morbidly obese individuals for whom TDM is advised to guide dosing interval adjustment. Our simulations suggest indeed that shortening the dosing interval from 8 to 4 weeks results in a lower proportion of morbidly individuals with concentrations predicted to be below the minimal thresholds at steady-state both for LA cabotegravir and rilpivirine.

Obese individuals often have associated comorbidities (eg diabetes, hypertension, dyslipidemia); however, the medications used to treat these diseases do not have any inducing effects on drug metabolizing enzymes and therefore are not expected to reduce cabotegravir or rilpivirine exposure [29].

A limitation of this study is that the obese population was between 20 and 50 years old; therefore, our analysis did not evaluate the combined effect of age and obesity. Furthermore, the population represented White obese individuals; however, it should be emphasized that our findings can be extrapolated to Black PWH because there are no ethnicity-related genetic polymorphisms in the enzymes metabolizing cabotegravir (UGT1A1) and rilpivirine (CYP3A4) and because the physiology is comparable between White and Black [13, 32]. Another limitation is that the PBPK model assumed that all the virtual subjects received the injection in the ventrogluteal muscles; however, real-life data have shown that the injected drug depot is not always deposited the muscle but sometimes also in the subcutaneous adipose tissue which can impact the drug release from the depot and thereby contribute to the variability in drug concentrations [33]. The probability of injecting LA cabotegravir/rilpivirine in the adipose tissue rather than the muscle is higher in obese PWH as the adipose layer is thicker compared to the one in normal weight individuals [34]. Thus, it is critical that injections are done with a longer needle in obese PWH.

## CONCLUSION

Morbidly obese PWH are at risk of presenting suboptimal trough concentrations of LA cabotegravir and rilpivirine not only after the first injection but also at steady-state, particularly, with the bimonthly administration. Therapeutic drug monitoring is advised to guide dosing interval adjustment. More clinical data are needed to confirm the findings of this modeling study and to evaluate whether monthly administration of LA cabotegravir/rilpivirine, notably in morbidly obese individuals, could prevent treatment failure in order to warrant changes in treatment guidelines. Furthermore, the impact of using a longer needle on the pharmacokinetics and response of LA cabotegravir/rilpivirine needs to be further evaluated in morbidly obese PWH individuals.

## Supplementary Data

Supplementary materials are available at *Clinical Infectious Diseases* online. Consisting of data provided by the authors to benefit the reader, the posted materials are not copyedited and are the sole responsibility of the authors, so questions or comments should be addressed to the corresponding author.

## Supplementary Material

ciae060_Supplementary_Data

## Notes

***Data availability statement.*** The data that support the findings of this study are available upon request to the corresponding author.

***Financial support***. This work was supported by the Swiss National Foundation (grant number 188504).
