# Supplementary material for: Effect of Obesity on the Exposure of Long-acting Cabotegravir and Rilpivirine: A Modeling Study
Source: Clin Infect Dis. 2024 Feb 3;79(2):477–86. doi: 10.1093/cid/ciae060 (PMC11327779; doi:10.1093/cid/ciae060)
Supplement: ciae060_Supplementary_Data [file ciae060_supplementary_data.docx]

Clinical Infectious Diseases

Supplementary Material

Effect of obesity on the exposure of long-acting cabotegravir and rilpivirine: a modelling study

Authors: Sara Bettonte^1, 2^, Mattia Berton^1, 2^, Felix Stader^3^, Manuel Battegay^1, 2^, and Catia Marzolini^1, 2, 4, 5^

Affiliations: 1 Division of Infectious Diseases and Hospital Epidemiology, Departments of Medicine and Clinical Research, University Hospital Basel, 4031 Basel, Switzerland

2 Faculty of Medicine, University of Basel, 4031 Basel, Switzerland

3 Certara UK Limited, Sheffield, UK

4 Department of Molecular and Clinical Pharmacology, University of Liverpool, L69 3GF Liverpool, UK

5 Service and Laboratory of Clinical Pharmacology, Department of Laboratory Medicine and Pathology, University Hospital Lausanne and University of Lausanne, Switzerland

Corresponding author:

Sara Bettonte, MS

Division of Infectious Diseases and Hospital Epidemiology

Departments of Medicine and Clinical Research

University Hospital Basel

Petersgraben 4

4031 Basel, Switzerland

E-mail: sara.bettonte@unibas.ch

Alternative corresponding author:

Catia Marzolini, PharmD, PhD

Division of Infectious Diseases and Hospital Epidemiology

Departments of Medicine and Clinical Research

University Hospital Basel

Petersgraben 4

4031 Basel, Switzerland

E-mail: catia.marzolini@usb.ch

**Methods**

Structure of the physiologically based pharmacokinetics (PBPK) model

Our in-house whole-body PBPK model developed in Matlab®2020a (MathWorks, Natick, MA) is constituted by 17 perfusion-limited compartments [1]. The in-house PBPK model was implemented with a framework describing the release of the long-acting (LA) drug from the depot into the blood stream. An additional compartment representing the injection site was added to the model structure (i.e., gluteus medius, gluteus minimus). Assuming that the LA injectable is administered in the interstitial space of the muscle [2], an additional compartment representing the depot was added into the interstitial space of the compartment representing the injection site. The final ordinary differential equation used to describe the release of the drug from the depot into the systemic circulation took into account the drug physicochemical properties and the physiology of the injection site [3].

PBPK model parameters

1. *Virtual obese population*

PBPK modelling allows to simulate unstudied clinical scenarios for which a cohort of virtual individuals is generated. In order to reproduce the physiology of the population of interest (i.e., obese), the PBPK model was informed with equations describing the physiological changes of an obese population with a body mass index (BMI) ranging from 18.5 up to 60 kg/m^2^ and aged between 20 and 55 years [4].

1. *Absorption phase*

To describe oral absorption, the compartmental absorption and transit (CAT) model constituted by stomach, duodenum, jejunum, ileum, and colon was implemented in the PBPK model [1, 5]. Published apparent permeability (P_app_) values measured *in vitro* or derived *in silico* [6] were converted into effective permeability in man (P_eff, man_) (equation 1) [1, 7]:

$P_{eff, man}={10}^{0.6795*\log\left( Papp \right)-0.3355}$ (1)

where P_app_ is expressed in 10^-6^ cm/s and P_eff,man_  in 10^-4^ cm/s.

By using the P_eff,man_ , the absorption clearance (Cl_ab_) was derived considering the structure of the different intestinal segment (equation 2) [1]:

${Cl}_{ab}= P_{eff, man}*2* \pi*r*Le*F_{villi}$ (2)

Where P_eff,man_ is expressed in 10^-4^ cm/s, r is the radius of each intestinal segment, Le is the length of each intestinal segment, and F_villi_ is the fold expansion factor for the villi surface area.

1. *Distribution phase*

During the drug model development, the fraction unbound in plasma (fu_p_) and the blood-to-plasma (B:P) ratio were added as initial parameters in the compound file and were subsequently used to determine the fraction of free drug available for drug metabolism. Additionally, in order to calculate the volume of distribution, the PBPK model used the equations of Rodgers and Rowland et al. [8-10] to determine the partition coefficients (tissue/plasma).

1. *Metabolism*

The abundances of each drug metabolizing enzymes were implemented in the virtual population [4]. Thus, in each drug files the enzyme parameters for each pathway responsible for the drug metabolism were added as intrinsic clearance (CL_int,e_) or as maximum metabolism rate for each enzyme (V_max,e_) and as Michaelis-Menten constant (K_m,e_). Subsequently, the model was sum up and scaled to total hepatic intrinsic clearance (CL_int, h, tot_) and the total gut intrinsic clearance (CL_int, g, tot_) was determined using equations 3 and 4, respectively [1]:

${CL}_{int,h,tot}= \sum\frac{V_{max, e}}{K_{m,e}}+{CL}_{int,e} \times{AB}_{h} \times MPPGL \times liver weight$ (3)

${CL}_{int,g,tot}= \sum\frac{V_{max, e}}{K_{m,e}}+{CL}_{int,e} \times{AB}_{g}$ (4)

Where AB_h_ and AB_g_ are the enzyme abundance in the liver and in the intestine and MPPGL is the microsomal protein per gram liver.

After deriving the total hepatic intrinsic clearance (CL_int, h, tot_), the liver well-stirred model was used to derive the total hepatic clearance (CL_hep_) which is expressed in L/h (equation 5) [1]:

${CL}_{hep}= \frac{Q_{li}\times{fu}_{BL}\times{CL}_{int,h,tot}}{Q_{li}+{fu}_{BL}\times{CL}_{int,h,tot}}$ (5)

Where Q_li_ is the liver blood flow, fu_BL_ is the fraction unbound in blood.

1. *Excretion*

The PBPK model does not consider the effect of active transport on drug elimination. Thus, the glomerular filtration rate (GFR) was used to scale the renal clearance.

PBPK modelling strategy

1. For each drug of interest, a drug model file was created. The physicochemical properties of the drug (e.g., density, solubility, octanol-water partition coefficient (log P)) alongside the parameters used to describe the absorption, distribution (e.g., fu_p_), metabolism (e.g., intrinsic clearance), and excretion (i.e., renal clearance) were added in each drug file.
2. In order to verify the ability of the model to correctly predict the distribution and the elimination phases, the drug models were verified against clinically observed data for intravenous administration (if available).
3. To verify the ability of the model to predict the absorption phase, the drug models were verified against clinically observed data after oral administration.

For cabotegravir and rilpivirine, the data used to develop the drug models are shown in Supplementary Table 1 and have been previously published by our group [11, 12]. Steps number 2 and 3 were conducted for normal weight (BMI 18.5-25 kg/m^2^) and obese (BMI >30 kg/m^2^) virtual individuals. The drug models were considered verified when the simulations were within 2-fold of clinically observed data as per PBPK model guidelines [13]. Specifically, the drug model of cabotegravir was considered verified as the simulation were within 1.25-fold of observed clinically observed data both for normal weight and obese individuals (Supplementary Table 2); while for rilpivirine the simulation were within 1.5-fold of clinically observed data for normal weight and obese individuals (Supplementary Table 2) [14].

Supplementary Table 1. Cabotegravir and rilpivirine parameters.

| Parameter | Unit | Cabotegravir | | Rilpivirine | |
| --- | --- | --- | --- | --- | --- |
| *Physicochemical properties* | | | | | |
| molecular weight | g/mol | 405.35 | [15] | 366 | [16] |
| log P |  | 1.58 | [15] | 4.32 | [16] |
| drug type |  | ma | [17] | mb | [18] |
| pK_a1_ |  | 7.71 | [15] | 3.26 | [16] |
| BP |  | 0.524 | [17] | 0.67 | [16] |
| fu_p_ |  | 0.006 | [15] | 0.003 | [16] |
| binding protein |  | albumin | [19] | albumin | [20] |
| density | g/mL | 1.6 | [21] | 1.3 | [22] |
| solubility | µmol/L | 29.1 | [23] | 50.5 | [24] |
| *Absorption* | | | | | |
| Caco-2 permeability | 10^-6^ cm/sec | 12.3 | retrograde calculation* | 12 | [16] |
| *Metabolism and Excretion* | | | | | |
| CYP3A4 CL_int_ | µl/min/pmol enzyme | - | | 2.04 | [16, 18] |
| UGT1A1 V_max_ | pmol/min/pmol enzyme | 16.1 | [17] | - | |
| UGT1A1 K_m_ | µM | 148 | [17] | - | |
| UGT1A9 V_max_ | pmol/min/pmol enzyme | 6.25 | [17] | - | |
| UGT1A9 K_m_ | µM | 90 | [17] | - | |
| Unspecified | µL/min/mg | - | | 93.2 | [18] |
| CL_renal_ | L/h | 0.008 | retrograde calculation* | - | |

**Abbreviations:** BP, blood-plasma-ratio; Cl_int_, intrinsic clearance; CL_renal_, renal clearance; CYP, cytochrome P-450; fu_p_, fraction unbound in plasma; K_m_, Michaelis-Menten constant; log P, octanol-water partition coefficient; ma, monoprotic acid; mb, monoprotic base; pk_a_, acid dissociation constant; UGT, uridine diphosphate-glucuronosyltransferase; V_max_, maximum velocity.

* cabotegravir Caco-2 permeability: derived using madin-darby canine kidney (MDCK) cell line value published by Reese et al. [25].

* cabotegravir CL_renal_: derived using in vitro–in vivo extrapolation (IVIVE).

Supplementary Table 2. Predicted versus observed data for cabotegravir and rilpivirine after oral administration.

|  | **Normal weight**  **(median BMI 24 kg/m^2^)** | | | **Obese**  **(median BMI 33 kg/m^2^)** | | | **Obese/normal weight** | | **Reference** |
| --- | --- | --- | --- | --- | --- | --- | --- | --- | --- |
|  | **Observed** | **Predicted** | **Ratio P/O** | **Observed** | **Predicted** | **Ratio P/O** | **Observed** | **Predicted** |  |
| **Cabotegravir 30 mg steady state** | | | | | | | | | |
| C_max_ [ng/mL] | - | 8,244 (50) | - | - | 6,346 (51) | - | - | 0.77 | [26] |
| C_τ_ [ng/mL] | 5,408 (48) | 5,426 (76) | 1.00 | 4,573 (50) | 4,189 (78) | 0.92 | 0.85 | 0.77 |  |
| AUC_0-τ_ [ng*h/mL] | - | 164,661 (61) | - | - | 126,646 (62) | - | - | 0.77 |  |
|  | **Normale weight**  **(median BMI 26 kg/m^2^)** | | | **Obese**  **(median BMI 34 kg/m^2^)** | | | **Obese/ normal weight** | |  |
| **Rilpivirine 25 mg steady state** | | | | | | | | | |
| C_max_ [ng/mL] | 160 (31) | 148 (38) | 0.93 | 148 (62) | 148 (33) | 1.00 | 0.92 | 1.00 | [14] |
| C_τ_ [ng/mL] | 75 (39) | 72 (29) | 0.96 | 89 (66) | 75 (25) | 0.84 | 1.19 | 1.04 |  |
| AUC_0-τ_ [ng*h/mL] | 2,333 (30) | 2,457 (30) | 1.05 | 1,981 (19) | 2,444 (27) | 1.23 | 0.85 | 0.99 |  |

**Abbreviations:** The data are expressed as geometric mean (CV%). AUC_0-τ_, area under the time-concentration curve to tau; BMI, body mass index; C_max_, peak concentration; C_τ_, trough concentration; O, observed; P, predicted.

**References**

1. Stader F, Penny MA, Siccardi M, Marzolini C. A comprehensive framework for physiologically based pharmacokinetic modelling in Matlab((R)). CPT Pharmacometrics Syst Pharmacol **2019**; 8(7): 444-59.

2. Chow TW, Wright MR, Hop C, Wong H. Evaluation of the predictive performance of physiologically based pharmacokinetic models for intramuscular injections of therapeutic proteins. Xenobiotica **2019**; 49(12): 1423-33.

3. Bettonte S, Berton M, Stader F, Battegay M, Marzolini C. Development of a physiologically based pharmacokinetic model to simulate the pharmacokinetics of intramuscular antiretroviral drugs (submitted). CPT: Pharmacometrics & Systems Pharmacology **2024**.

4. Berton M, Bettonte S, Stader F, Battegay M, Marzolini C. Repository describing the anatomical, physiological, and biological changes in an obese population to inform physiologically based pharmacokinetic models. Clin Pharmacokinet **2022**; 61(9): 1251-70.

5. Yu LX, Amidon GL. A compartmental absorption and transit model for estimating oral drug absorption. Int J Pharm **1999**; 186(2): 119-25.

6. Gertz M, Harrison A, Houston JB, Galetin A. Prediction of human intestinal first-pass metabolism of 25 CYP3A substrates from in vitro clearance and permeability data. Drug Metab Dispos **2010**; 38(7): 1147-58.

7. Sun D, Lennernas H, Welage LS, et al. Comparison of human duodenum and Caco-2 gene expression profiles for 12,000 gene sequences tags and correlation with permeability of 26 drugs. Pharm Res **2002**; 19(10): 1400-16.

8. Rodgers T, Leahy D, Rowland M. Physiologically based pharmacokinetic modeling 1: predicting the tissue distribution of moderate-to-strong bases. J Pharm Sci **2005**; 94(6): 1259-76.

9. Rodgers T, Rowland M. Physiologically based pharmacokinetic modelling 2: predicting the tissue distribution of acids, very weak bases, neutrals and zwitterions. J Pharm Sci **2006**; 95(6): 1238-57.

10. Rodgers T, Rowland M. Mechanistic approaches to volume of distribution predictions: understanding the processes. Pharm Res **2007**; 24(5): 918-33.

11. Bettonte S, Berton M, Stader F, Battegay M, Marzolini C. Management of drug-drug interactions between long-acting cabotegravir and rilpivirine and comedications with inducing properties: a modeling study. Clin Infect Dis **2023**; 76(7): 1225-36.

12. Bettonte S, Berton M, Stader F, Battegay M, Marzolini C. Intramuscular cabotegravir and rilpivirine concentrations after switching from efavirenz-containing regimen. Br J Clin Pharmacol **2023**; 89(12): 3618-28.

13. Shebley M, Sandhu P, Emami Riedmaier A, et al. Physiologically based pharmacokinetic model qualification and reporting procedures for regulatory submissions: a consortium perspective. Clin Pharmacol Ther **2018**; 104(1): 88-110.

14. Berton M, Bettonte S, Stader F, et al. Antiretroviral drug exposure and response in obese and morbidly obese people with HIV: a study combining modelling and Swiss HIV Cohort data. Clin Infect Dis **2023** [epub ahead or print].

15. Taskar K, Patel P, Cozens S, et al. Utilization of physiologically based pharmacokinetic modelling (PBPK) to predict the effect of UGT enzyme inhibition and induction on the systemic exposure of cabotegravir. 20th International Workshop on Clinical Pharmacology of HIV Hepatitis & Other Antiviral Drugs (2020) 2020, Noordwijk, the Netherlands, abstract.

16. Rajoli RK, Back DJ, Rannard S, et al. Physiologically based pharmacokinetic modelling to inform development of intramuscular long-acting nanoformulations for HIV. Clin Pharmacokinet **2015**; 54(6): 639-50.

17. Bowers GD, Culp A, Reese MJ, et al. Disposition and metabolism of cabotegravir: a comparison of biotransformation and excretion between different species and routes of administration in humans. Xenobiotica **2016**; 46(2): 147-62.

18. Center for drug evaluation and research. Clinical pharmacology and biopharmaceutics review(s). Addendum to Ondqa biopharmaceutics review Available at: <https://www.accessdata.fda.gov/drugsatfda_docs/nda/2011/202022Orig1s000ClinPharmR.pdf>. Accessed October 2023.

19. Shaik JSB, Ford SL, Lou Y, et al. A phase 1 study to evaluate the pharmacokinetics and safety of cabotegravir in patients with hepatic impairment and healthy matched controls. Clin Pharmacol Drug Dev **2019**; 8(5): 664-73.

20. U.S. Food and Drug Administration. Edurant product label. Available at: <https://www.accessdata.fda.gov/drugsatfda_docs/label/2011/202022s000lbl.pdf>. Accessed October 2023.

21. ChemSpider. Experimental data Cabotegravir: density. Available at: <http://www.chemspider.com/Chemical-Structure.30829503.html?rid=3f61d1d5-97fe-4d34-9bd9-a3f9d12d75fb&page_num=0> Accessed October 2023.

22. ChemSpider. Experimental data Rilpivirine: density. Available at: <http://www.chemspider.com/Chemical-Structure.4953643.html>. Accessed October 2023.

23. Center for Drug evaluation and Research. Product quality reviews Available at: <https://www.accessdata.fda.gov/drugsatfda_docs/nda/2021/212887Orig1s000,212888Orig1s000ChemR.pdf>. Accessed October 2023.

24. Kommavarapu P, Maruthapillai A, Palanisamy K, Sunkara M. Preparation and characterization of rilpivirine solid dispersions with the application of enhanced solubility and dissolution rate. Beni-Suef University Journal of Basic and Applied Sciences **2015**; 4(1): 71-9.

25. Reese MJ, Bowers GD, Humphreys JE, et al. Drug interaction profile of the HIV integrase inhibitor cabotegravir: assessment from in vitro studies and a clinical investigation with midazolam. Xenobiotica **2016**; 46(5): 445-56.

26. Elliot E, Polli JW, Patel P, et al. Efficacy, safety and pharmacokinetics by BMI category in phase 3/3b cabotegravir and rilpivirine long-acting trials. J Infect Dis **2023**; [epub ahead of print].
